# IRF4 regulates myeloid-derived suppressor cells expansion and function in *Schistosoma japonicum*-infected mice

**DOI:** 10.1186/s13071-024-06543-8

**Published:** 2024-11-28

**Authors:** Lu Zhou, Peibin Lin, Guorong Deng, Lengshan Mo, Cansheng Hong, Zhihan Jiang, Yiqiang Zhu, Yi Zhao, Yanwei Qi, Tengfei Hu, Qianlian Wu, Jian Zhang, Qingqing Li, Quan Yang

**Affiliations:** 1 Affiliated Qingyuan Hospital, Qingyuan People’s Hospital, Guangzhou Medical University, Qingyuan, 511518 China; 2 Department of Pathogenic Biology and Immunology, Sino-French Hoffmann Institute, School of Basic Medical Sciences, Guangzhou Medical University, Guangzhou, 511436 China; 3 The Second Affiliated Hospital, The State Key Laboratory of Respiratory Disease, Guangdong Provincial Key Laboratory of Allergy & Clinical Immunology, Guangzhou Medical University, Guangzhou, 510260 China; 4 The Affiliated TCM Hospital of Guangzhou Medical University, Guangzhou, 510130 China; 5 School of Biomedical Engineering, Guangzhou Medical University, Guangzhou, 511436 China

**Keywords:** *Schistosoma japonicum*, Interferon regulatory factor 4, Myeloid-derived suppressor cell, Infection

## Abstract

**Background:**

Interferon regulatory factor 4 (IRF4) is a crucial member of the IRF family of transcription factors and is pivotal in orchestrating the body’s defense against tumors and infections by modulating the differentiation and functionality of immune cells. The role of IRF4 in mice during *Schistosoma japonicum* infection, as well as the effects of IRF4 deficiency on myeloid-derived suppressor cells (MDSCs), remains inadequately understood.

**Methods:**

Hematoxylin and eosin staining was used to evaluate the pathological damage in different organs of mice following infection with *S. japonicum*. Flow cytometry was employed to study the effect of IRF4 on the proliferation and function of myeloid-derived suppressor cells (MDSCs) in *S. japonicum*-infected mice.

**Results:**

Knockout of IRF4 in myeloid cells significantly mitigated pathological damage to the liver and lungs in mice infected with *S. japonicum*. Knockout of IRF4 in myeloid cells also inhibited the expansion and functionality of MDSCs by downregulating programmed death ligand 2 (PD-L2) expression and interleukin-1 alpha (IL-1α) secretion in mice infected with *S. japonicum*. Mechanistic studies revealed that IRF4 deficiency inhibited the expansion and function of MDSCs and that this inhibition was mediated by the STAT3 and AKT signaling pathways. Also, IRF4 myeloid knockout promoted the expansion of T cells in *S. japonicum*-infected mice, but had no significant effect on B cell aggregation.

**Conclusions:**

Overall, these findings highlight the importance of IRF4 in regulating MDSCs and their impact on tissue damage during *S. japonicum* infection, providing valuable insights into potential therapeutic targets for managing the pathological consequences of this parasitic infection.

**Graphical Abstract:**

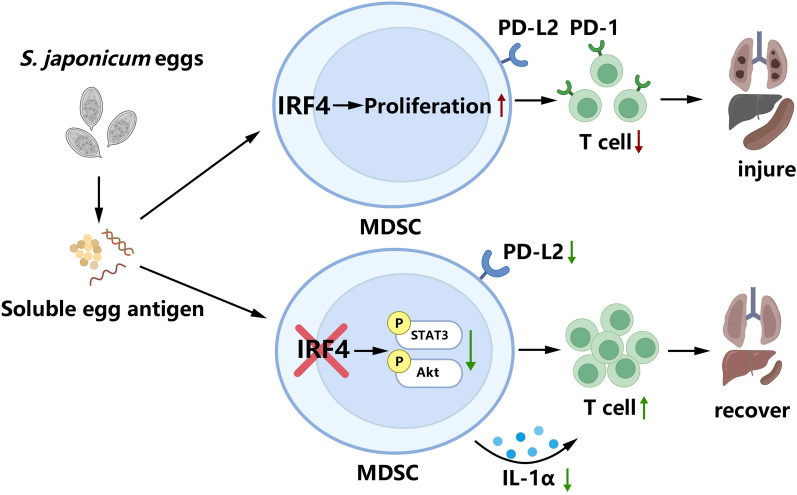

**Supplementary Information:**

The online version contains supplementary material available at 10.1186/s13071-024-06543-8.

## Background

Schistosomiasis is a neglected tropical disease caused by parasitic flatworms belonging to the genus *Schistosoma*, and a significant public health challenge in Africa, Asia, the Caribbean and South America [1]. Six different *Schistosoma* species can cause schistosomiasis in humans, with *S. japonicum* being the most common in China [2, 3]. Following *Schistosoma* infection, the initial symptoms may include fever, myalgia, exhaustion, nonproductive cough and bloody diarrhea [3, 4]. If not treated in time, the infection can lead to severe complications and even death. After passing through the portal vein-vena cava shunt, the eggs can be transported to lung capillaries, inducing granuloma formation in the area around the alveoli, possibly resulting in fibrosis, which can further result in cor pulmonale and pulmonary hypertension [5]. A high-intensity, established and active infection of *Schistosoma* triggers a robust immunopathological response centered on the formation of granulomas around the eggs deposited in the spleen and liver. This process leads to inflammation and fibrosis, resulting in significant enlargement of both organs [6, 7], particularly in children [8]. A granuloma is a specialized type of inflammatory lesion that forms around foreign substances, as part of the host's immune response. The formation of granulomas and subsequent fibrosis occurs swiftly after egg deposition in tissues. Typically, *Schistosoma* eggs collect in the liver and intestines of the host, where they cause inflammation, leading to granuloma and fibrosis of the host tissue.

The immune system’s reaction to soluble egg antigen (SEA) is the primary driver of schistosomiasis pathology, and the relationship between *Schistosoma* and the host immune system is extremely intricate [9, 10]. The main immune response post-*Schistosoma* infection initially involves Th1 cells, which secrete cytokines such as interferon gamma (IFN-γ) and interleukin (IL)-2, mediating cellular immunity and contributing to parasite clearance. However, as the parasite matures and begins to produce eggs, the immune response shifts towards Th2, characterized by the production of cytokines such as IL-4, IL-5, and IL-13, which promote humoral immunity and eosinophil/mast cell activation [11]. The delicate balance between Th1 and Th2 responses is crucial for host resistance to *Schistosoma* infection, as it enables the host to effectively control parasite proliferation while avoiding excessive tissue damage caused by unregulated inflammation. This balance is essential for maintaining a healthy immunological milieu that can both eliminate the parasite and promote tissue repair and healing [12, 13]. Using a mouse model of schistosomiasis infection facilitates exploration of the important factors of granuloma formation and increase current understanding of immunopathological mechanisms.

The regulation of the host’s innate and adaptive immune responses is crucial for the initiation and maintenance of *Schistosoma* infection [14], necessitating the involvement of antigen-presenting cells (APCs), including dendritic cells (DCs) and macrophages. SEA has been extensively studied for its ability to modulate the function of APCs, particularly DCs and macrophages, and has been shown to condition DCs to promote a type 2 immune response by skewing their cytokine secretion profile towards IL-4, IL-5 and IL-13, which are hallmarks of Th2-mediated immunity [15, 16]. Additionally, SEA has been demonstrated to induce an alternatively activated phenotype in macrophages, characterized by increased expression of arginase-1 and Ym1/2, as well as enhanced phagocytic activity and production of anti-inflammatory cytokines like IL-10 and tumor growth factor beta (TGF-β) [17, 18]. It has also been demonstrated that excretory/secretory (E/S) products of *Schistosoma* might partially trigger macrophage cytokine response [19]. These changes contribute to the regulation of excessive inflammation and the maintenance of parasite persistence within the host. In addition, a variety of cells and molecules are involved in the immunopathogenesis of *Schistosoma* infection. Myeloid-derived suppressor cells (MDSCs) represent a diverse population of immature cells that exhibit potent immunosuppressive properties. Based on the expression levels of Ly6C and Ly6G markers to distinguish myeloid populations, MDSCs can be categorized into two main types: polymorphonuclear MDSCs (PMN-MDSCs) and monocytic MDSCs (M-MDSCs). The two subsets have different biological functions and different regulatory mechanisms [20, 21]. MDSCs have been shown to proliferate and accumulate in diseased tissues after parasite infection, a process which significantly suppresses the host immune response and regulates excessive inflammation [22]. Our previous research indicated a significant accumulation of MDSCs in the spleen, bone marrow (BM) and lymph nodes of mice infected with *S. japonicum* [23].

Interferon regulatory factor 4 (IRF4), a transcription factor belonging to the IRF family of transcription factors, is predominantly expressed in various immune cells, including lymphoid cells, macrophages, DCs and monocytes [24–27]. Its expression can be induced by multiple signaling pathways, such as the Toll-like receptor (TLR) signal in macrophages, T cell receptor (TCR) in T cells, B cell receptor (BCR) in B cells, as well as IL-4 and CD40 signals in B cells [24, 28]. IRF4 plays a crucial role in regulating adaptive immunity, facilitating the differentiation, affinity maturation and functionality of macrophages, DCs, T cells and B cells [29]. IRF4 is critically involved in tumorigenesis and the immune system, with its dysregulated expression closely linked to the development of autoimmune diseases, lymphomas, multiple myelomas and other malignancies [30, 31]. Recent studies have further implicated IRF4 in modulating the immunosuppressive capacity of MDSCs, suggesting that its downregulation during tumorigenesis may contribute to MDSC expansion [32]. While the precise role of IRF4 in the differentiation and function of MDSCs during schistosomiasis, particularly the disease caused by *S. japonicum*, remains understudied, intriguing parallels can be drawn from other experimental models of parasitic infections. For example, a recent study investigating pulmonary inflammation in human and murine schistosomiasis caused by *S. mansoni* highlighted the critical function of type 2 dendritic cells (DC2s) and their reliance on IRF4 for proper development and function [33]. In the present study, the use of IRF4 conditional knockout (KO) mice (Cre^+^CD11c∆Irf4 model) provided insights into the importance of IRF4 in DC2-mediated immune responses. Although in this study we did not directly examine IRF4^flox/flox^/LysM^Cre+^(IRF4 KO) mice in the context of *S. japonicum* infection, our results underscore the potential impact of IRF4 deficiency on the overall immune response to schistosome infections. Given the essential role of IRF4 in DC2 differentiation and function, we hypothesized that IRF4 KO mice would likely exhibit altered immune responses during schistosomiasis, particularly affecting Th2-mediated inflammation, which is a hallmark of this parasitic disease. The absence of IRF4 could lead to impaired DC2 development or function, resulting in reduced Th2 cytokine production and potentially influencing parasite clearance or disease progression. However, to conclusively determine the outcomes of IRF4 KO mice in schistosomiasis models infected with *S. japonicum*, further experimental studies are warranted.

In the study reported here, we examined the impact of IRF4 myeloid knockout on infection outcomes across various organs, along with the progression of MDSC activation and differentiation in mice infected with *S. japonicum*.

## Methods

### Mice

The IRF4 conditional mutant mice (*IRF4*^*flox/flox*^; Stock No. 009380) and *LysM-Cremice* (B6N. 129P2 [B6] Lyz2tm1 [cre] Ifo/J; Stock No: 018956) were initially acquired from the Jackson Laboratory (Bar Harbor, ME, USA). These mice were bred with a C57B/L6 background for maintenance purposes. Female C57BL/6 mice, aged between 6 and 8 weeks, were acquired from the Animal Experimental Center at Sun Yat-Sen University (Guangzhou, China). To generate mice IRF4-KO mice, we crossed *LysM-Cre* mice with IRF4*flox/flox* mice, and subsequently established cohorts by mating F1 *IRF4flox*/+; *Cre*+ mice with littermate *IRF4flox*/+; *Cre*- mice. The Laboratory Animal Centre, Guangzhou Medical University, maintains a pathogen-free microenvironment for all mice.

### Parasites and infection

*Schistosoma japonicum* cercariae that had been originally acquired from* Oncomelania hupensis* snails that were naturally infected were obtained from the China Institute of Parasitic Diseases in Shanghai, China. *IRF4*^*flox/flox*^*/LysM-Cre*^*−*^ (WT) and *IRF4*^*flox/flox*^*/LysM-Cre*^+^ (IRF4-KO) female mice aged 6–8 weeks were infected with 40 ± 5 cercariae through the abdominal skin. The experiments on animals were conducted in strict adherence to the regulations governing the management of experimental animals, with utmost attention paid towards minimizing animal distress.

### Reagents and antibodies

The RPMI 1640 medium (Cat. no. C11875500BT), penicillin–streptomycin solution (Cat. no. 15140122), carboxyfluorescein succinimidyl ester (CFSE; Cat. no. C34554) and fetal bovine serum (FBS) serum (Cat. no. 10099141) were acquired from Invitrogen (Thermo Fisher Scientific, Waltham, MA, USA). The red blood cells (RBCs) lysis buffer (Cat. no. C3702) was obtained from Beyotime Biotechnology (Shanghai, China). Brefeldin A (Cat. no. B5936), dimethyl sulfoxide (DMSO) (Cat. no. D2650), ionomycin (Cat. no, I3909) and phorbol 12-myristate 13-acetate (PMA; Cat. no. P1585) were purchased from Sigma-Aldrich (St. Louis, MO, USA). Fluorescein-conjugated anti-mouse antibodies (CD11b-PE-Cy7 [M1/70], Cat. no. 25-0112-82; Gr1-FITC [RB6-8C5], Cat. no. 11-5939-86; Gr1-PE [RB6-8C5], Cat. no. MA1-83934); Ly6C-PECP-Cy5.5 [HK1.4], Cat. no. 45-5932-82); CD4-PECP-Cy5.5 [RM4-4], Cat. no. 116012; IL-1α-PE [ALF-161], Cat. no. 12-7011-82; CD8-PE [53–6.7], Cat. no. MA1-10304) and their respective isotype controls for the antibodies were acquired from eBioscience (San Diego, CA, USA). Fluorescein-conjugated anti-mouse antibodies (IL-6-APC [pMP5-20F3], Cat. no. 504507; Ly6G-PE [1A8], Cat. no. 551461; PD-L2-APC [TY25], Cat. no. 560086; NF-κB p65 [pS529], Cat. no. 558423), their corresponding isotype controls and the Annexin V-PE Apoptosis Kit (Cat. no. 559763) were obtained from BD Biosciences (San Jose, CA, USA). Fluorescein-conjugated anti-mouse antibodies (MHC-II-FITC [AF6-120.1], Cat. no. 116405; MHC-II-PECP-Cy5.5 [AF6-120.1], Cat. no. 116415; CD3-APC-Cy7 [145-2C11], Cat. no. 100330; CD19-APC-Cy7 [6D5], Cat. no. 115529; CD69-APC [H1.2F3], Cat. no. 104513; GM-CSF-PECP-Cy5.5 [MP1-22E9], Cat. no. 505409; PD-L1-BV421 [10F.6G2], Cat. no. 124315); CD11c-PECP-Cy5.5 [N418], Cat. no. 117328; F4/80-APC-Cy7 [BM8], Cat. no. 123118; IL-10-APC [JES5-16E3], Cat. no. 505010; IFN-γ-APC [XMG1.2], Cat. no. 505809; IL-4-PE ([1B11], Cat. no. 504103; IL-17- PE-Cy7 [TC11-18H10.1], Cat. no. 506921; p-STAT3-BV421 [Tyr705], Cat. no. 651009t), their corresponding isotype controls and purified anti-mouse CD3 (17A2, Cat. no. 100202)/CD28 (37.51, Cat. no. 122002) antibody were obtained from BioLegend (San Diego, CA, USA). Fluorescein-conjugated anti-mouse antibodies (p-AKT-APC, Cat. no. 17-9715-41) and the corresponding isotype control were obtained from Thermo Fisher Scientific (Waltham, MA, USA).

### Histology studies

For the histology studies, the mice were euthanized and the thorax and trachea immediately injected with 0.01 M phosphate-buffered saline (PBS, pH 7.4) to ensure adequate perfusion of the liver, spleen and lungs, which were subsequently treated with 10% formalin, followed by embedding in Paraffin and sectioning. The sections were stained using the traditional hematoxylin–eosin (H&E) method and then examined under a light microscope. Histological images were analyzed and the granuloma area was quantified using ImageJ software (National Institutes of Health, Bethesda, MD, USA) by an observer who was blind to the treatment history. The size of granulomas was expressed as the mean area in square micrometers ± standard deviation (SD).

### Isolation of lymphocytes

After the mice were euthanized, various organs were harvested. The lungs removed from the mice were first lavaged with PBS to remove any peripheral blood, following which the lung tissue was fragmented using ophthalmic scissors and digested in a digestive buffer containing collagenase IV and DNase I at 37 °C for 30 min. The lung tissue and spleen were mechanically homogenized using a sterile syringe piston, followed by filtration through a 100-μm filter. To obtain a cell suspension, the cell mixture was treated with RBC lysis buffer for 10 min and then rinsed twice with HBSS buffer. The liver removed from mice was digested using the Miltenyi Biotec Liver Dissociation Kit (Bergisch Gladbach, Germany) to dissociate the tissue into a cell suspension. A density gradient centrifugation technique was then employed using the Ficoll-Hypaque density gradient medium (Dakewe, Shenzhen, China) to separate and isolate immune cells from the cell fluid. The femurs and tibias of the mice were used to collect the mouse BM. A complete RPMI 1640 culture medium was used to resuscitate the lymphocytes for further experiments.

### Magnetic bead sorting

The Myeloid-Derived Suppressor Cell Isolation Kit (Miltenyi Biotective, Cat no. 130-094-538) was used to sort mouse MDSCs. First, 100 million lymphocytes were suspended in sorting buffer twice, and each time the supernatant was discarded following centrifugation. FcR-blocking antibody and biotin-conjugated Gr-1 antibody were added successively to the suspension and the suspension incubated for 10 min at 4 °C. Cells were subsequently incubated with anti-biotin microbeads for 15 min at 4 °C, followed by washing and resuspension in sorting buffer. Before separation, the MS column (Miltenyi Biotec, Cat no. 130-042-201) was washed in separation buffer, following which the cells conjugated with antibodies were introduced into the column and subjected to two rounds of washing using sorting buffer. Subsequently, the magnetic field was deactivated, leading to the collection of target cells. Flow cytometry (FCM) was used to analyze the purity of the target cells.

### Cell surface staining

For cell surface staining, cells were first washed twice in PBS, subjected to a 30-min blocking process using PBS buffer supplemented with 1% bovine serum albumin (BSA) and then labeled with conjugated antibodies that specifically target cell surface antigens. This labeling procedure was carried out in a dark environment at 4 °C for 30 min. These antigens included CD11b, Gr1, CD11c, MHC-II, F4/80, Ly6C, Ly6G, CD3, CD4, CD8, PD-L1, PD-L2 and CD69. The lymphocytes that had been stained were washed twice in a buffer solution (PBS with 1% BSA) and then resuspended in 300 μl of the same buffer. To ensure accuracy, isotype-matched controls were incorporated into each staining procedure. The stained lymphocytes were analyzed by FCM on a Beckman Coulter instrument, and the obtained results were analyzed using CytoExpert 2.3 software (Beckman Coulter Inc., Brea, CA, USA).

### Cell intracellular cytokine staining

Single-cell suspensions from various organs of mice were exposed to a combination of 20 ng/ml PMA and 1 μg/ml ionomycin for 5 h at 37 °C in the presence of a 5% CO_2_ atmosphere. Brefeldin A (10 g/ml; Sigma Shanghai, Shanghai, China) was introduced during the last 4 h of the incubation. Following washing in PBS, the cells were subjected to staining using conjugated antibodies specific to cell surface antigens (CD11b and Gr1); this staining process occurred in darkness at 4 °C for up to 30 min. The cells were then washed twice with PBS, fixed using paraformaldehyde solution at a concentration of 4% and permeabilized overnight at 4 °C in PBS buffer containing saponin (Sigma-Aldrich, St Louis, MO, USA), BSA and NaN_3_. The cells were then stained utilizing conjugated antibodies specific to intracellular cytokine antigens, under dark conditions at 4 °C for 30 min. These cytokine antigens included IL-1α, GM-CSF, IL-6, IL-10, IL-4, IL-17 and IFN-γ. After two washes with washing buffer (PBS supplemented with BSA at an amount equaling 1%), the stained lymphocytes were resuspended in 300 μl washing buffer. In cases where transcription factors needed to be stained, no prior cell stimulation step was necessary. The next step involved staining the cells using conjugated antibodies that targeted both transcriptional factors and cytokines; these specific antigens included p-p65, pSTAT1, pATAT3 and p-AKT. Isotype-matched cytokine controls were incorporated into each individual staining protocol. FCM was used to analyze stained lymphocytes, and CytoExpert 2.3 software was used to analyze the results ((Beckman Coulter Inc.).

### Annexin V staining

The cells were first labeled with a fluorescence-labeled antibody specific for MDSCs (CD11b^+^Gr1^+^) at 4 ˚C for 30 min. They were the washed and stained with annexin V-PE as per the instructions provided by the manufacturer (BD Pharmingen, Franklin Lakes, NJ, USA).

### Quantitative real-time PCR

The MDSCs were subjected to RNA extraction using TRIzol reagent (Invitrogen Life Technologies, Thermo Fisher Scientific)) according to the manufacturer's instructions, which included DNase treatment. A SuperScript III Reverse Transcriptase Kit (Qiagen, Valencia, CA, USA) was used to convert 0.5 μg of total RNA into complementary DNA (cDNA). The expression levels of transcripts were normalized against β-actin transcripts using the 2^−△△Ct^ method with relative quantity (RQ); the primer sequences are given in Table 1.

**Table 1:** Sequences of primers used in the quantitative real-time PCR assays

Gene	Forward primer (5′-3′)	Reverse primer (5′-3′)
*IRF4*	agaccagacttgcaagctct	caccaaagcacagagtcacc
*gp91*	gcaggaaaggaacaatgcca	gggtgttcacttgcaatggt
*Arg1*	ctacccactgcctttgaagc	ggtatcacaggacagcaggt
*NOS2*	ccccgctactactccatcag	ccactgacacttcgcacaaa
*p40*	atcaacctactgctgtcccc	gacccatggagccaactttg
*p47*	ccaagtcttcaagccagcag	ggagtcagattcgggcacta
*P67*	gctctcatgcatgccaagaa	catggccttgtcgatcttgg
*IL-1α*	ccgtgttgctgaaggagttg	gtgcacccgactttgttctt
*GM-CSF*	ttggaagcatgtagaggcca	cgcccttgagtttggtgaaa
*IL-6*	agacttccatccagttgcct	caggtctgttgggagtggta
*IL-10*	ggtgagaagctgaagaccct	acaccttggtcttggagctt
*Caspase3*	cagccaacctcagagagaca	acaggcccatttgtcccata
*Caspase8*	aactgcgtttcctaccgaga	ccttgttcctcctgtcgtct
*FAS*	gaggcccattttgctgtcaa	ttcccttctgtgcatgaggt
*Bcl-xL*	cgtggaaagcgtagacaagg	gctgcattgttcccgtagag
*HIF1α*	ctcaccagacagagcaggaa	aagggagccatcatgttcca
*COX2*	accgagtcgttctgccaata	gcttgatttagtcggcctgg
*S100A8*	aggaaatcaccatgccctct	tgagatgccacacccacttt
*S100A9*	gccaacaaagcaccttctca	ttccttcttgctcagggtgt
*β-actin*	actgctctggctcctagcac	acatctgctggaaggtggac

### Enzyme-linked immunosorbent assay measurement of SEA-specific immunoglobulin A and immunoglobulin G

The levels of immunoglobulin (Ig)G and IgM in the serum of mice that were infected were measured by enzyme-linked immunosorbent assay (ELISA) . Serum samples were extracted from the inner canthal veins of infected WT and IRF4-KO mice. SEA was diluted to 80 μl/ml with sterile PBS, and a 100-μl aliquot was added to each well of the ELISA plate, followed by incubation at 4 °C for 14–18 h. After the incubation, the samples were washed 5 times in PBST, and diluted serum was added to each well (100 μl/well) and the plate incubated at 37 °C for 2 h. Following this second incubation the samples were washed again 5 times in PBST, following which horseradish peroxidase (HRP) enzymic antibody was added to each well at the appropriate dilution (100 μl/well) and the plate was incubated at 37 °C for 1 h. Then, the samples were washed 10 times with PBST and the substrate solution was added to each well (100 μl/well), ensuring no exposure to light for a period ranging from 5 to 30 min. The reaction was terminated by adding a diluted sulfuric acid solution (10%) to each well (50 μl/well). The results were detected using an enzyme labeling instrument and read at a wavelength of 450 nm using a microplate reader (Moder ELX-800; BioTek, Winooski, VT, USA).

### Statistics

The unpaired t-test was used to analyze and compare the means of different groups. In cases where the data did not follow a normal distribution, a nonparametric test was employed for analysis and comparison. Data analysis was conducted using the GraphPad Prism v6.02 (GraphPad Software Inc., San Diego, CA, USA) and SPSS Statistics version 17.0 (SPSS IBM Corp., Armonk, NY, USA) software packages. A significance level of *P* < 0.05 was considered to be statistically significant.

## Results

### Effects of IRF4 myeloid konckout on pathological changes in organs in mice infected with *S. japonicum*

**Fig. 1:**
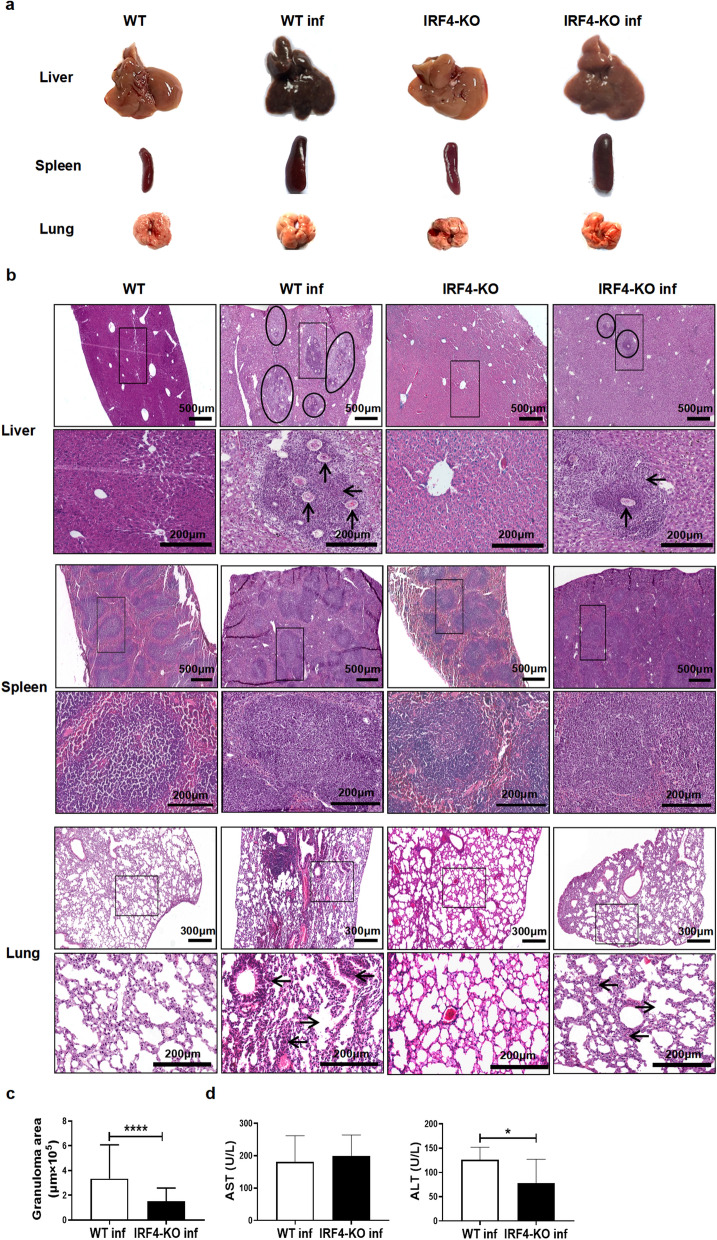
Effects of IRF4 myeloid knockout on pathological changes in organs of mice infected with *Schistosoma japonicum*. **a**-**d** WT mice and IRF4-KO mice were infected percutaneously with 40 ± 5 cercariae and sacrificed at 6–7 weeks after infection. The tissues of the liver, spleen and lung were harvested for further study. **a** Representative pictures of liver, spleen and lung of naive (WT and IRF4-KO) and infected (WT inf and IRF4-KO inf) mice. **b** Representative pictures of H&E staining of liver, spleen and lung in the four experimental groups of mice: the circle indicates granulomas, the left-pointing arrow indicates leukocyte infiltration, the upwards-pointing arrow indicates the eggs of *S. japonicum *and the right-pointing arrow indicates alveolar dilation. **c** Area of granuloma in infected WT and IRF4-KO mice. A representative of three independent experiments with 3 mice per group is shown. Data are expressed as the mean ± SD of 64–73 granulomatous areas. **d** Expression of ALT and AST in serum of infected WT and IRF4-KO mice. Data are presented as mean ± SD of 8 mice. Asterisks indicate a statistically significant difference at *P < 0.05 and ****P < 0.0001, compared with the corresponding control (unpaired *t*-test). ALT, alanine aminotransferase; AST, aspartate aminotransferase; H&E, hematoxylin and eosin stain; IRF4, interferon regulatory factor 4; IRF4-KO, IRF4 knockout mice strain; SD, standard deviation; WT, wild-type

To investigate the effect of the IRF4 gene on mice infected with *S. japonicum*, we infected WT and IRF4-KO mice with *S. japonicum* cercariae through abdominal skin and then sacrificed the mice 6–7 weeks after infection for testing. The results indicated that post infection with *S. japonicum*, the liver became brownish and hardened, with numerous gray-white egg nodules, and the spleen enlarged significantly; in contrast, no remarkable changes were observed in the lungs (Fig. 1a). Notably, compared to the liver of *S. japonicum*-infected WT mice, the liver of infected IRF4-KO mice appeared lighter in color, although no significant differences were evident the coloration of the spleen and lungs (Fig. 1a). Also, the ratio of liver, spleen and lung weight, respectively, to body weight remained similar between infected IRF4-KO and infected WT mice (Additional file 1: Figure S1). Histological analysis using H&E staining showed that there were egg granulomas in the liver of mice infected with *S. japonicum*, accompanied by extensive leukocyte infiltration. In contrast, no significant differences were observed in the spleens between the two groups of infected mice (Fig. 1b, c). The H&E results of the lungs revealed that the lung tissue structure of uninfected mice was clear, while the infected mice exhibited alveolar dilation and inflammatory cell infiltration. Compared with infected WT mice, infected IRF4-KO mice had relatively few alveolar fusion and inflammatory cells (Fig. 1b). We also observed that the liver granuloma area of infected IRF4-KO mice was significantly smaller than that in infected WT mice (Fig. 1c; *t*-test: WT inf versus IRF4-KO inf: *t*(135) = 5.302, *P *< 0.0001). To further investigate the impact of IRF4 deletion on the liver function of mice infected with *S. japonicum*, we measured the expression levels of aspartate aminotransferase (AST) and alanine aminotransferase (ALT) in the serum of infected mice of both the WT and IRF4-KO strains. ALT levels in infected IRF4-KO mice were found to be significantly lower than those in infected WT mice (Fig. 1d; *t*-test: WT inf versus IRF4-KO inf: *t*(14) = 2.416, *P *= 0.03), suggesting that liver injury was more severe in infected WT mice. These findings suggest that the absence of IRF4 may confer a protective effect against egg-induced tissue damage in mice infected with *S. japonicum*.

### Effects of IRF4 myeloid knockout on MDSCs, DCs and macrophages in mice infected with *S. japonicum*

**Fig. 2:**
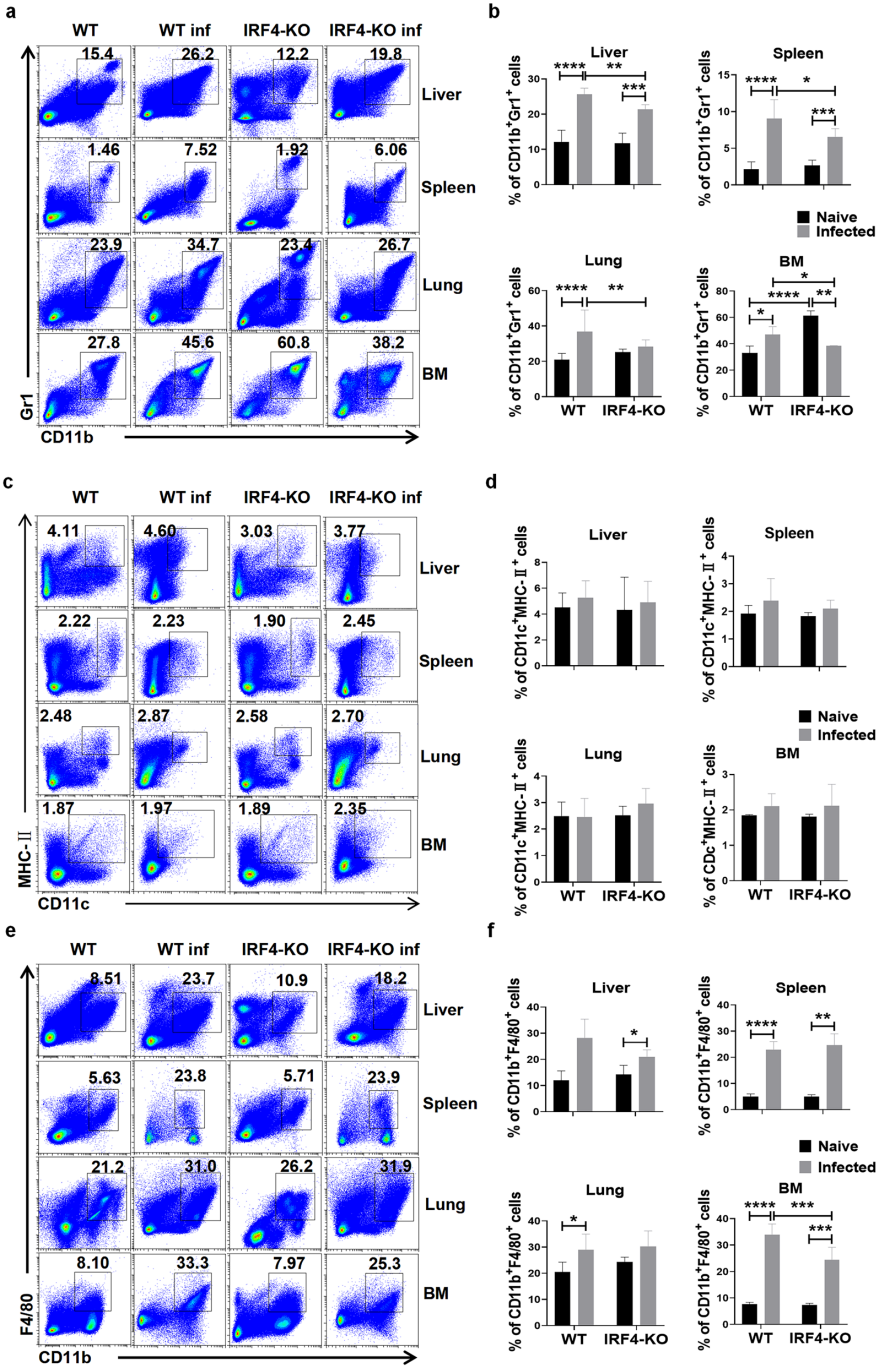
Effects of IRF4 myeloid knockout on MDSCs, DCs and macrophages in mice infected with *Schistosoma japonicum*. **a**-**f** WT mice and IRF4-KO mice were infected percutaneously with 40 ± 5 cercariae and sacrificed at 6–7 weeks after infection. Single cell suspensions from liver, spleen, lung and BM of naive (WT and IRF4-KO) and infected (WT inf and IRF4-KO inf) mice were isolated. The percentages of MDSCs (CD11b^+^Gr1^+^ cells) (**a**,** b**), DCs (CD11c^+^MHC-II^+^ cells) (**c**, **d**) and macrophages (CD11b^+^F4/80^+^ cells) (**e**, **f**) in the liver, spleen, lung and BM were determined by flow cytometry. Representative results (left) and the statistical representation (right) are shown. Data are expressed as the mean ± SD of 3–32 mice. Asterisks indicate a statistically significant difference at *P < 0.05, **P < 0.01, ***P < 0.001 and ****P < 0.0001, compared with the corresponding control (unpaired *t*-test). BM Bone marrow; DC, dendritic cells; IRF4, interferon regulatory factor 4; IRF4-KO, IRF4 knockout mice strain; MDSCs, myeloid-derived suppressor cells; SD, standard deviation; WT, wild-type

To explore the effect of IRF4 on the existence and distribution of MDSCs (CD11b^+^Gr1^+^ cells), DCs (CD11b^+^CD11c^+^ cells) and macrophages (CD11b^+^F4/80^+^ cells) in different organs of the mice infected with *S. japonicum*, we established both naive and *S. japonicum*-infected WT and IRF4-KO mouse models (*n* = 4 models) and used FCM to detect the expression of MDSCs, DCs and macrophages in the liver, spleen, lung and BM of these four groups of mice. The results showed that the proportion of MDSCs was significantly higher in different organs of mice infected with *S. japonicum* than in their organ counterparts of naive mice (Fig. 2a; *t*-test: WT Naive versus WT Infected: Liver: *t*(8)=8.618, *P *< 0.0001; Spleen: *t*(14)=7.369, *P* < 0.0001; Lung: *t*(62)=7.136, *P* < 0.0001; BM: *t*(10)=4.155, *P* = 0.002. IRF4-KO Naive versus IRF4-KO Infected: Liver: *t*(8) = 6.72, *P* = 0.0001; Spleen: *t*(11) = 5.54, *P* = 0.0002). However, comparison of BM from uninfected IRF4-KO mice and infected IRF-KO mice showed that the proportion of MDSCs in the BM of infected IRF4-KO mice decreased significantly (*t*-test: IRF4-KO Naive versus IRF4-KO Infected: *t*(9)=10.23, *P* < 0.0001), suggesting potential differential effects of IRF4 on MDSCs in the BM of uninfected versus infected mice. Compared with the proportion of MDSCs in the studied organs in infected WT mice, the proportion of MDSCs in the liver, spleen, lung and BM of infected IRF4-KO mice was significantly lower (Fig. 2a, b; *t*-test: WT Infected versus IRF4-KO Infected: Liver: *t*(10)=4.474, *P* = 0.0012; Spleen: *t*(15)=2.725, *P* = 0.0157; Lung: *t*(26)=3.252, *P* = 0.0032; BM: *t*(8)=2.388, *P* = 0.044). Regarding DCs, there was no significant difference in the proportion between naive mice and *S. japonicum*-infected mice (*t*-test: WT Naive versus WT Infected: Liver: *t*(7)=0.9182, *P* = 0.3891; Spleen: *t*(4)=0.9441, *P* = 0.3986; Lung: *t*(5)=0.05605, *P* = 0.9575; BM: *t*(5)=1.313, *P* = 0.2462. IRF4-KO Naive versus IRF4-KO Infected: Liver: *t*(6)=0.4037, *P* = 0.7004; Spleen: *t*(4)=1.392, *P* = 0.2365; Lung: *t*(5)=1.145, *P* = 0.304; BM: *t*(4)=0.8371, *P* = 0.4496), as well as no significant difference between infected WT mice and infected IRF4-KO mice (Fig. 2c, d; *t*-test: WT Infected versus IRF4-KO Infected: Liver: *t*(8) = 0.404, *P* = 0.6968; Spleen: *t*(4) = 0.5963, *P* = 0.5996; Lung: *t*(6) = 1.105, *P* = 0.3114, BM: *t*(5 = 0.002322, *P* = 0.9982). In addition, the proportion of macrophages in the liver, spleen, lung and BM of *S. japonicum*-infected mice was significantly higher than that of naive mice. Compared with infected WT mice, the proportion of macrophages in BM of IRF4-KO infected mice was significantly reduced, but there was no significant difference in the liver, spleen and lung (Fig. 2e, f; *t*-test: WT Infected versus IRF4-KO Infected: Liver: *t*(7) = 1.656, *P* = 0.1418; Spleen: *t*(7) = 0.6988, *P* = 0.5072; Lung: *t*(26) = 0.5446, *P* = 0.5907; BM: *t*(17) = 4.485, *P* = 0.0003). These results suggest that IRF4 may be an important transcription factor regulating the differentiation and accumulation of MDSCs in different organs of *S. japonicum*-infected mice.

### Effects of IRF4 myeloid knockout on MDSC subsets and functional molecules in mice infected with *S. japonicum*

**Fig. 3:**
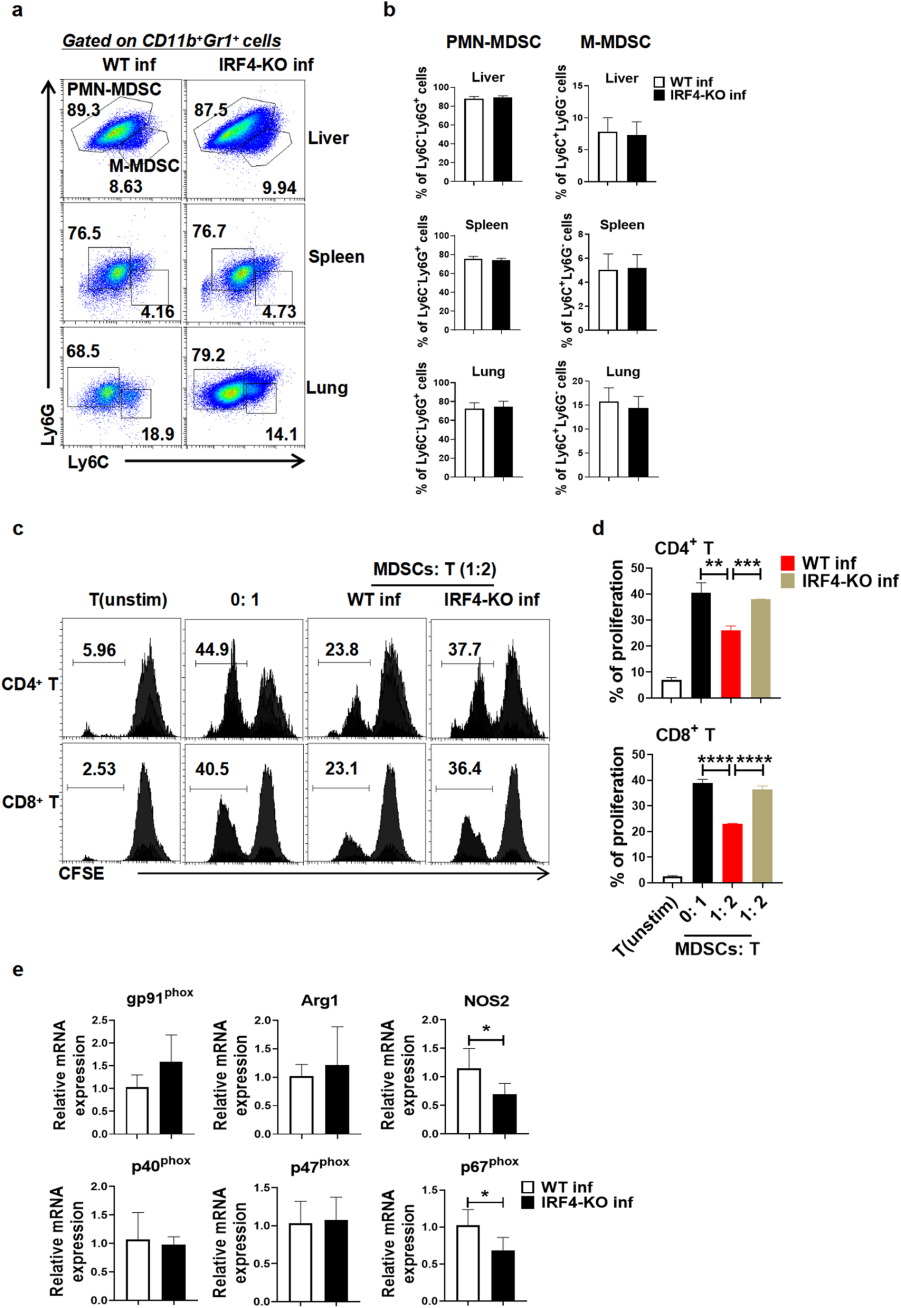
Effects of IRF4 myeloid knockout on MDSC subsets and functional molecules in mice infected with *Schistosoma japonicum*. **a**-**e** WT mice and IRF4-KO mice were infected percutaneously with 40 ± 5 cercariae and sacrificed at 6–7 weeks after infection. Single cell suspensions from liver, spleen and lung of infected WT (WT inf) and infected IRF4-KO (IRA-KO inf) mice were isolated. **a**, **b** Proportions of MDSC subtypes in the liver, spleen, and lung as evaluated by flow cytometry. Data are expressed as the mean ± SD of 4–6 mice. **c**, **d** MDSCs were sorted and purified by magnetic beads and then co-cultured with ConA-stimulated CD3^+^ T cells at a 1:2 ratio for 3 days. T cell proliferation was measured by CFSE dilution. Unstimulated. T cells were used as the negative control. After 3 days, the proliferation of T cells was analyzed by flow cytometry. **e** Spleen MDSCs of infected WT and infected IRF4-KO mice were separated by magnetic beads, and the expression of *gp91*^*phox*^, *Arg1*, *NOS2*, *p40*^*phox*^, *p47*^*phox*^ and *p67*^*phox*^ in MDSCs was detected by qRT-PCR. Asterisks indicate a statistically significant difference at *P < 0.05, **P < 0.01, ***P<0.001 and ****P<0.0001, compared with the corresponding control (unpaired *t*-test). CFSE, Carboxyfluorescein succinimidyl ester; IRF4, interferon regulatory factor 4; IRF4-KO, IRF4 knockout mice strain; MDSCs, myeloid-derived suppressor cells; M-MDSCs, monocytic MDSCs; PMN-MDSCs, polymorphonuclear MDSCs; qRT-PCR, quantitative real-time PCR; SD, standard deviation; WT, wild-type

As shown in Fig. 2, while IRF4 knockout had a pronounced effect on MDSCs in infected mice, as evidenced by significant changes in their proportions across different organs, notable alterations were also observed in macrophages, particularly in the BM, suggesting that IRF4 plays a multifaceted role in regulating myeloid cell populations during *S. japonicum* infection. To further explore the effect of IRF4 on MDSCs of mice infected with *S. japonicum*, we detected the subsets of MDSCs in infected mice. As shown in Fig. 3a, b, no significant difference was observed in the proportions of PMN-MDSCs and M-MDSCs in the liver, spleen and lung of infected WT mice and infected IRF4-KO mice. To assess the impact of IRF4 on the functional properties of MDSCs induced by *S. japonicum* infection, we performed a functional assay. The results of this assay demonstrated that MDSCs from *S. japonicum-*infected mice significantly suppressed T cell proliferation, whereas this inhibitory effect was significantly reduced in infected IRF4-KO mice (Fig. 3c, d; *t*-test: CD4^+^ T: 0: 1 versus WT inf: *t*(4) = 5.87, *P* = 0.0042; WT inf versus IRF4-KO inf: *t*(4) = 11.26, *P* = 0.0004. CD8^+^ T: 0: 1 versus WT inf: *t*(4) = 19.5, *P* < 0.0001; WT inf versus IRF4-KO inf: *t*(4) = 16.89, *P* < 0.0001). In order to explore the way through which IRF4 mediates the immunosuppressive activity of MDSCs in mice infected with *S. japonicum*, we detected the expression of MDSC-related functional genes. The results of the quantitative real-time PCR (qRT-PCR) assays revealed that, compared with the spleen MDSCs from infected WT mice, the expressions of *NOS2* and *p67*^*phox*^ were significantly reduced in the spleen MDSCs of infected IRF4-KO mice ( *t*-test: WT inf versus IRF4-KO inf: *NOS2*: *t*(9) = 2.715, *P* = 0.0238; *p67*^*phox*^: *t*(9)=2.867, *P* = 0.0186) and that the expressions of *gp91*^*phox*^, *Arg1*, *p40*^*phox*^ and *p47*^*phox*^ were not significantly different (Fig. 3e; *t*-test: WT inf versus IRF4-KO inf: *gp91*^*phox*^: *t*(4) = 1.493, *P* = 0.2097; *Arg1*: *t*(4) = 0.5002, *P* = 0.6432; *p40*^*phox*^: *t*(4) = 0.3031, *P* = 0.7769; *p47*^*phox*^: *t*(10)=0.2852, *P* = 0.7813).

### Effects of IRF4 myeloid knockout on the expression of MDSC surface molecules and cytokines in mice infected with *S. japonicum*

**Fig. 4:**
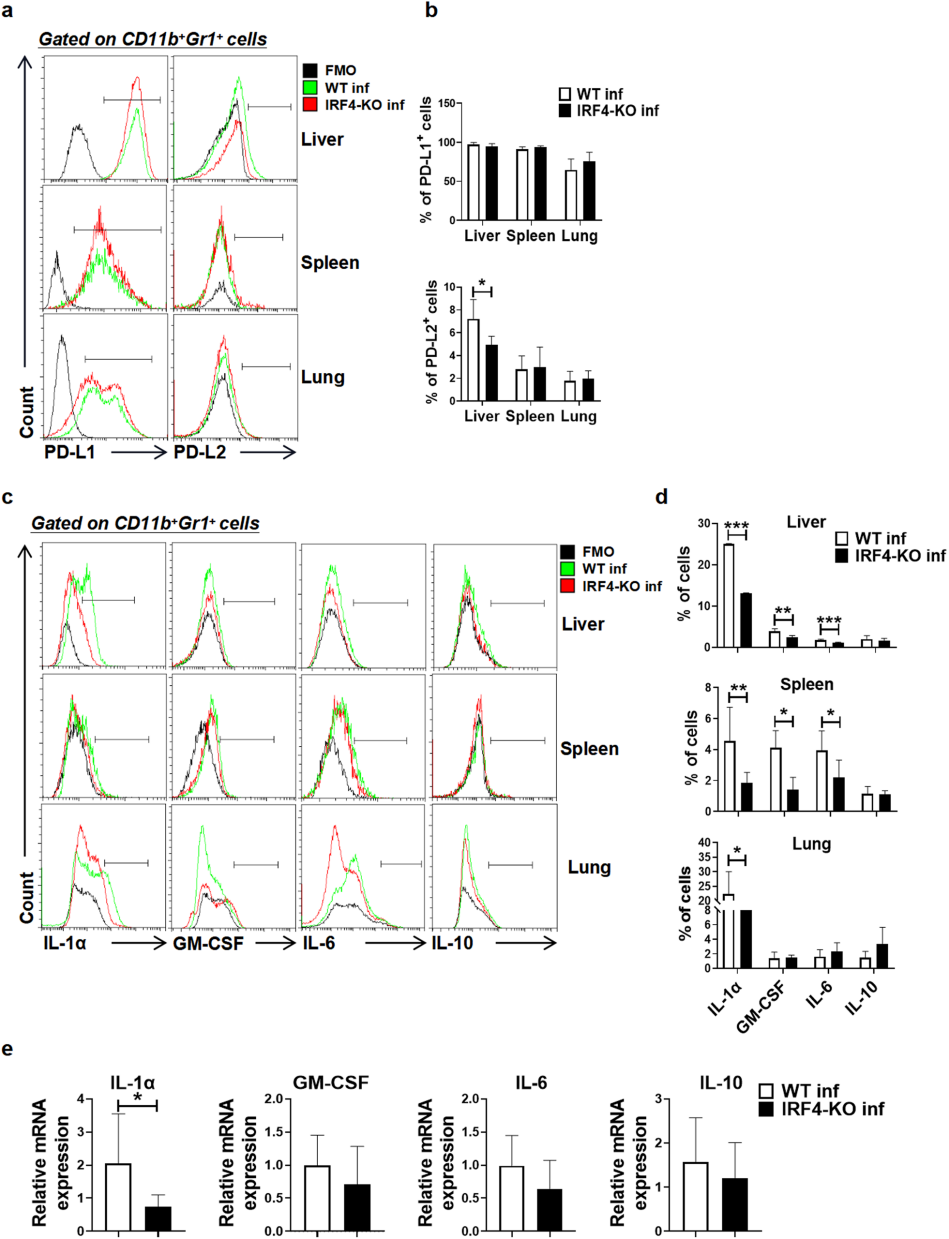
Effects of IRF4 myeloid knockout on the expression of MDSC surface molecules and cytokines in mice infected with *Schistosoma japonicum*. **a**-**e** WT mice and IRF4-KO mice were infected percutaneously with 40 ± 5 cercariae and sacrificed at 6–7 weeks after infection. Single cell suspensions from liver, spleen and lung of infected WT (WT inf) and infected IRF4-KO (IRF4-KO inf) mice were isolated. **a**, **b** Phenotypic analysis of CD11b^+^Gr1^+^ cells from the liver, spleen and lung of mice by different fluorescence-labeled antibodies to mouse surface markers, including PD-L1 and PD-L2. Data are expressed as the mean ± SD of 4–6 mice. **c**, **d** Single cell suspensions of liver, spleen and lungs from mice were stimulated with PMA and ionomycin. The expressions of IL-1α, GM-CSF, IL-6 and IL-10 were detected in MDSCs by FACS analysis. Data are expressed as the mean ± SD of 3–16 mice. Representative results (left) and the corresponding the statistical representation (right) are shown. **e** Spleen MDSCs of infected WT and infected IRF4-KO mice were separated by magnetic beads, and the expressions of IL-1α, GM-CSF, IL-6 and IL-10 in MDSCs were detected by qRT-PCR. Asterisks indicate a statistically significant difference at *P < 0.05, **P < 0.01 and ***P < 0.001, compared with the corresponding control (unpaired *t*-test). FACS, Fluorescence-activated cell sorting; GM-CSF, granulocyte-macrophage colony-stimulating factor; IRF4, interferon regulatory factor 4; IRF4-KO, IRF4 knockout mice strain; IL, interleukin; MDSCs, myeloid-derived suppressor cells; PD-L1/L2, programmed death receptor ligand 1/2; qRT-PCR, quantitative real-time PCR; SD, standard deviation; WT, wild-type

In order to further explore the effect of IRF4 on the function of MDSCs in mice infected with *S. japonicum*, we analyzed the expression of surface molecules and cytokines of MDSCs by flow cytometry and qRT-PCR. The results showed that there was no significant difference in programmed death receptor ligand 1 (PD-L1) expression in MDSCs in the liver, spleen and lung between infected WT mice and infected IRF4-KO mice (*t*-test: WT inf versus IRF4-KO inf: Liver: *t*(8) =1.108, *P* = 0.3002; Spleen: *t*(8)=1.468, *P* = 0.1803; Lung: *t*(8)= 1.292, *P* = 0.2324). Regarding PD-L2, PD-L2 expression in MDSCs in the liver of infected IRF4-KO mice was significantly lower than that in MDSCs in the liver of infected WT mice ( *t*-test: WT inf versus IRF4-KO inf: *t*(7) = 2.438, *P* = 0.0449), but there was no significant difference between the spleen and lung (Fig. 4a, b; *t*-test: WT inf versus IRF4-KO inf: Spleen: *t*(7) = 0.1889, *P* = 0.8555; Lung: *t* (8) =0.3355, *P* = 0.7459). For cytokines, compared with infected WT mice, the expression of IL-1α, GM-CSF and IL-6 in the liver and spleen of infected IRF4-KO mice decreased significantly (*t*-test: WT inf versus IRF4-KO inf: Liver: IL-1α: *t*(5)=109.2, *P* < 0.0001; GM-CSF: *t*(8)=0.3707, *P* = 0.006; IL-6: *t*(8) = 6.247, *P* = 0.0002. Spleen: IL-1α: *t*(17) = 3.706, *P* = 0.0018; GM-CSF: *t*(14) = 5.706, *P* < 0.0001; IL-6: *t*(11)=2.617, *P* = 0.024), but only the expression of IL-1α decreased significantly in lung (Fig. 4c, d; *t*-test: WT inf versus IRF4-KO inf: *t*(20)=2.9, *P* = 0.0088). The results of the qRT-PCR assays showed that the expression of *IL-1α* in the spleen of infected IRF4-KO mice was significantly lower than that in infected WT mice (Fig. 4e; *t*-test: WT inf versus IRF4-KO inf: *t*(16) = 2.536, *P* = 0.022), which was consistent with the results of the flow cytometry. These results suggest that IRF4 can regulate the function of MDSCs during *S. japonicum* infection.

### IRF4 myeloid knockout inhibits MDSC differentiation through the STAT3 and AKT signal pathways after *S. japonicum* infection

**Fig. 5:**
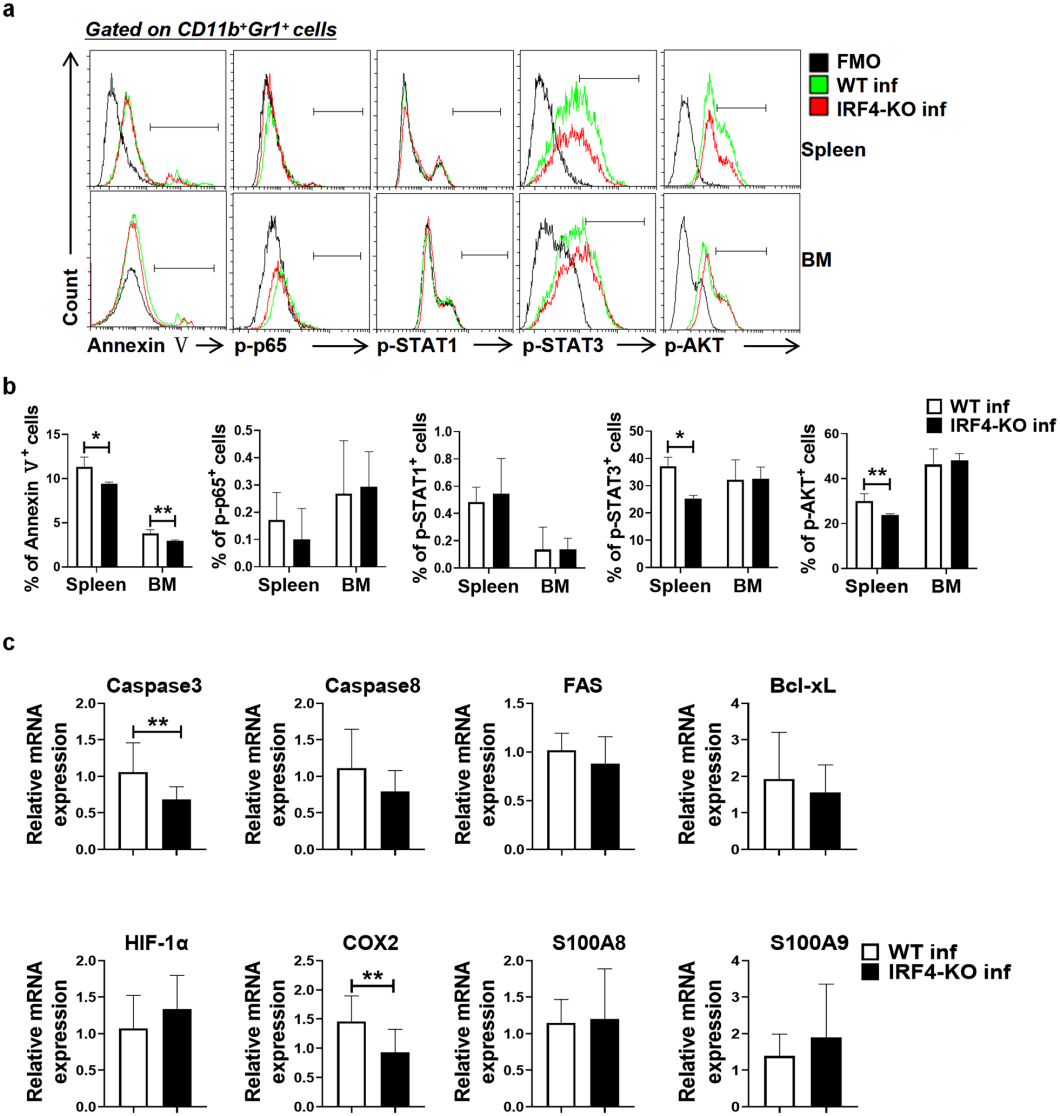
IRF4 myeloid knockout inhibits MDSC differentiation through the STAT3 and AKT signal pathways after *Schistosoma japonicum* infection. **a**-**c** WT mice and IRF4-KO mice were infected percutaneously with 40 ± 5 cercariae and sacrificed at 6–7 weeks after infection. Single cell suspensions from the spleen and BM of infected WT (WT inf) and infected IRF4-KO (IRF4-KO inf) mice were isolated. **a**-**b** Single cell suspensions from the spleen and BM were isolated from both WTinf and infected TLR7-KO mice for flow cytometry analysis. Data are expressed as the mean ± SD of 3–6 mice. **c** MDSCs were purified from the spleen of WT inf and IRF4-KO inf, and gene expression was determined by qRT-PCR. Asterisks indicate a statistically significant difference at *P < 0.05, **P < 0.01, compared with the corresponding control (unpaired *t*-test). BM, Bone marrow; FMO, fluorescence minus one; IRF4, interferon regulatory factor 4; IRF4-KO, IRF4 knockout mice strain; IL, interleukin; MDSCs, myeloid-derived suppressor cells; mRNA, messenger RNA; qRT-PCR, quantitative real-time PCR; SD, standard deviation; TLR, Toll-like receptor TLR; WT, wild-type

To investigate the mechanisms underlying the decrease in MDSCs following IRF4 myeloid knockout during *S. japonicum* infection, we employed FCM and qRT-PCR. Our results, as depicted in Fig. 5a, b, revealed that the expressions of p-STAT3, p-AKT and annexin V in the spleen MDSCs of infected IRF4-KO mice were significantly reduced compared to their expression in infected WT mice (Fig. 5a, b; *t*-test: WT inf versus IRF4-KO inf: p-STAT3: *t*(8)=6.607, *P* = 0.0002; p-AKT: *t*(6)=3.722, *P* = 0.0098; Annexin V: *t*(5)=2.92, *P* = 0.033). This finding suggested that IRF4 positively regulated the activation of the STAT3 and AKT signaling pathways in spleen MDSCs during *S. japonicum* infection. p-STAT3 and p-AKT are the activated forms of STAT3 and AKT, respectively, which are crucial for regulating cellular processes such as proliferation, survival and differentiation in MDSCs. Previous studies have shown that the activation of STAT3 and AKT promotes the immunosuppressive functions and survival of MDSCs [34–36]. Therefore, the observed decrease in p-STAT3 and p-AKT expression in IRF4-KO mice indicated that IRF4 is essential for maintaining these signaling pathways, which in turn supports the immunosuppressive activity and survival of MDSCs. Furthermore, the results of the qRT-PCR assays showed that the expression of *Caspase-3* and *COX2* in MDSCs in the spleen of infected WT mice was significantly lower than that in the spleen in infected IRF4-KO mice (Fig. 5c; *t*-test: WT inf versus IRF4-KO inf: *Caspase-3*: *t*(22) = 2.979, *P*=0.0069; *COX2*: *t*(19) = 2.931, *P*=0.0086), but that there was no significant difference in the expression of other genes (Fig. 5c; *t*-test: WT inf versus IRF4-KO inf: *Caspase8*: *t*(21)=1.779, *P* = 0.0898; *FAS*: *t*(10) = 1.03, *P* = 0.3274; *Bcl-xL*: *t*(20) = 0.8422, *P* = 0.4096; *HIF-1α*: t(9)=0.9379, *P* = 0.3728; *S100A8*: *t*(15) = 0.2018, *P* = 0.8428; *S100A9*: *t*(16) = 0.9584, *P* = 0.3521). Our findings demonstrated that IRF4 can regulate spleen MDSCs through positive regulation of the STAT3 and AKT signaling pathways during *S. japonicum* infection.

### Effect of IRF4 myeloid knockout on T cells in mice infected with *S. japonicum*

**Fig. 6:**
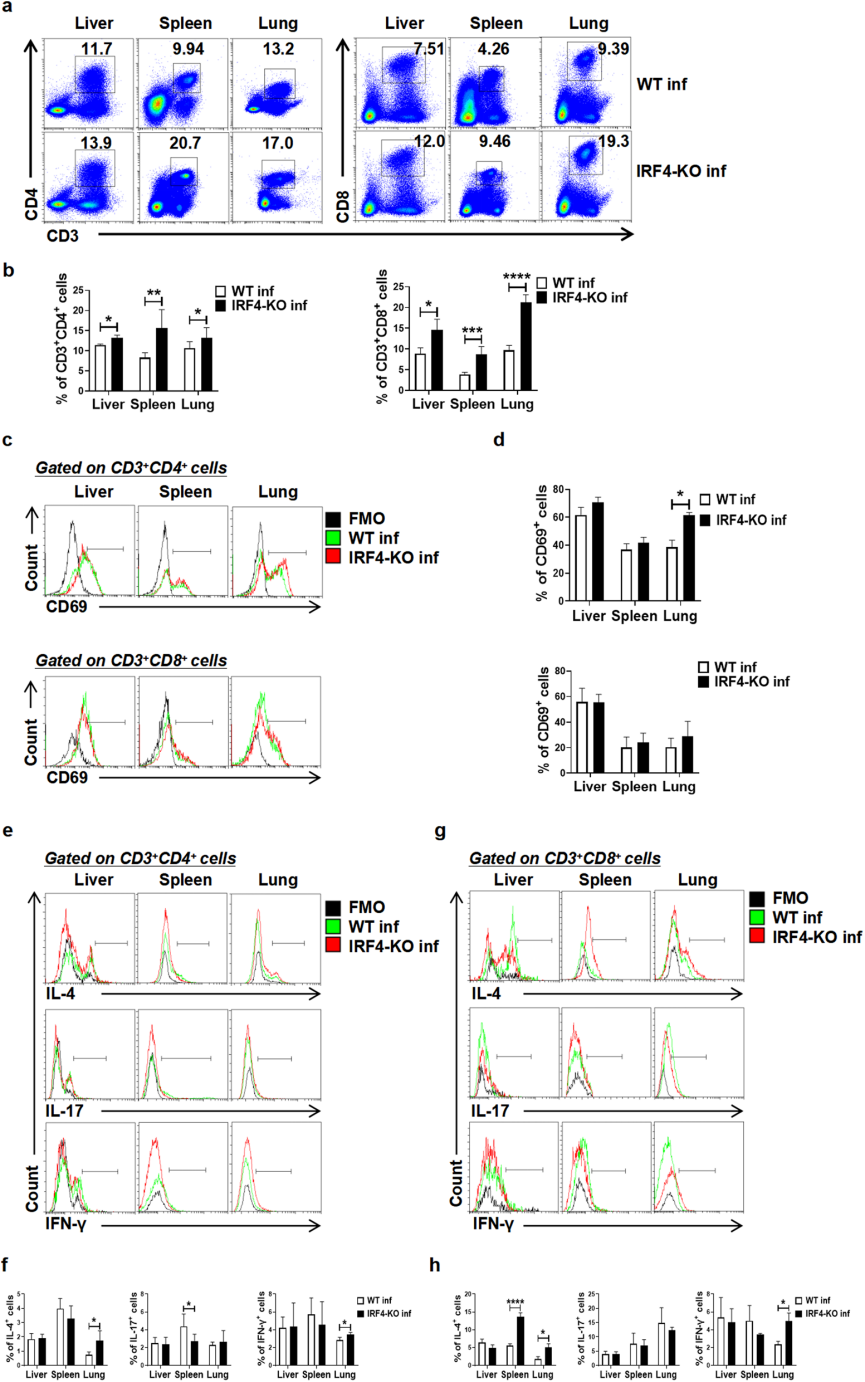
Effect of IRF4 myeloid knockout on T cells in mice infected with *Schistosoma japonicum*. **a**-**h** WT and IRF4-KO mice were infected percutaneously with 40 ± 5 cercariae and sacrificed at 6–7 weeks after infection. Single cell suspensions from liver, spleen and lung of infected WT (WT inf) and infected IRF4-KO (IRF4-KO inf) mice were isolated. **a**, **b** Proportions of the CD3^+^CD4^+^ T cells and CD3^+^CD8^+^ T cells in the liver, spleen and lung were evaluated by flow cytometry. Data are expressed as the mean ± SD of 3–8 mice. **c**-**h** Flow cytometry was used to detect the expression of CD69 in CD3^+^CD4^+^ T cells and CD3^+^CD8^+^ T cells, and the secretion of IL-4, IL-17 and IFN-γ. Data are expressed as the mean ± SD of 3–6 mice. Asterisks indicate a statistically significant difference at *P < 0.05, **P < 0.01, ***P<0.001 and ***P<0.0001, compared with the corresponding control (unpaired *t*-test), FMO, Fluorescence minus one; IFN, interferon; IRF4, interferon regulatory factor 4; IRF4-KO, IRF4 knockout mice strain; IL, interleukin; SD, standard deviation; WT, wild-type

To further explore the effect of IRF4 myeloid knockout on T cells in infected mice, we detected the percentage, activation level and cytokine production of CD3^+^CD4^+^ T cells and CD3^+^CD8^+^ T cells from these mice. As shown in Fig. 6a, b, the percentages of CD3^+^CD4^+^ T cells and CD3^+^CD8^+^ T cells in the liver, spleen and lung of infected WT mice were significantly higher than their counterparts in infected IRF4-KO mice (*t*-test: WT inf versus IRF4-KO inf: CD3^+^CD4^+^ T cells: Liver: *t*(4)=4.058, *P* = 0.0154; Spleen: *t*(9)=3.491, *P* = 0.0068; Lung: *t*(11)=2.285, *P* = 0.0417. CD3^+^CD8^+^ T cells: Liver: *t*(4)=3.366, *P* = 0.0281; Spleen: *t*(8)=5.859, *P* = 0.0004; Lung: *t*(6)=10.83, *P* < 0.0001). In addition, compared with infected WT mice, the expression of CD69 molecules related to T cell activation in infected IRF4-KO mice was only significantly increased in lung CD3^+^CD4^+^ T cells (Fig. 6c, d; *t*-test: WT inf versus IRF4-KO inf: *t*(7) = 7.344, *P* = 0.0002). Regarding cytokines, after IRF4 myeloid knockout in infected mice, the levels of IL-4 and IFN-γ secreted by CD3^+^CD4^+^ T cells and CD3^+^CD8^+^ T cells in the lungs were significantly increased (Fig. 6e-h; *t*-test: WT inf versus IRF4-KO inf: CD3^+^CD4^+^ T cells: IL-4: *t*(8)=3.38, *P* = 0.0096; IFN-γ: *t*(8)=3.707, *P*=0.006. CD3^+^CD8^+^ T cells: IL-4: *t*(8)=5.632, *P* = 0.0005; IFN-γ: *t*(6)=6.008, *P* = 0.001). Similarly, the level of IL-4 secreted by splenic CD3^+^CD8^+^ T cells increased significantly (Fig. 6e-h; *t*-test: WT inf versus IRF4-KO inf: *t*(8)=15.76, *P* < 0.0001). These results suggested that IRF4 myeloid knockout may increase immunological response by decreasing infection-induced MDSCs in *S. japonicum* infection, which would lessen the inhibition of T cell proliferation and activation.

### Effect of IRF4 myeloid knockout on B cells in mice infected with *S. japonicum*

**Fig. 7:**
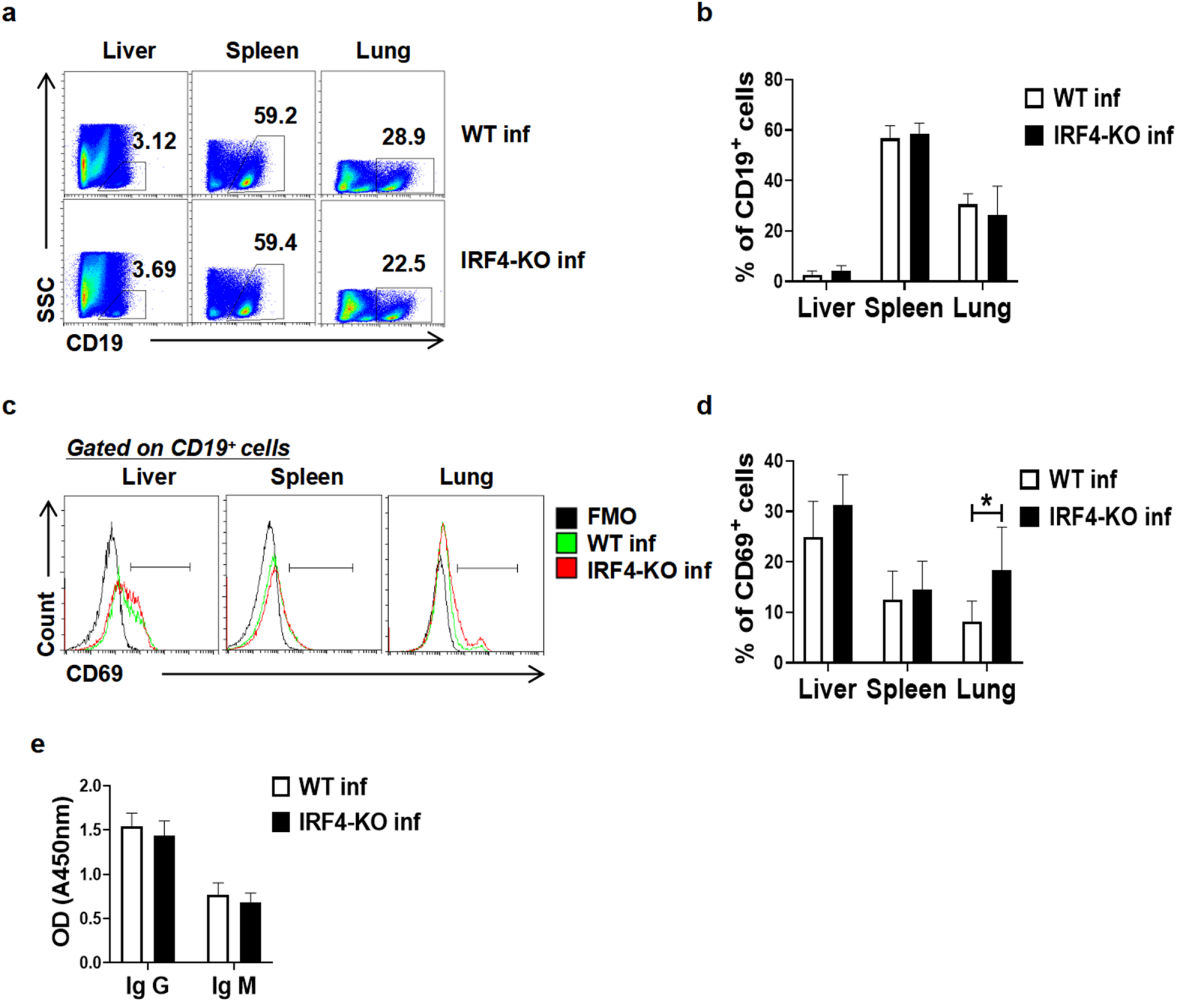
Effect of IRF4 myeloid knockout on B cells in mice infected with *Schistosoma japonicum*. **a**–**e** WT mice and IRF4-KO mice were infected percutaneously with 40 ± 5 cercariae and sacrificed at 6–7 weeks after infection. Single cell suspensions from liver, spleen and lung of infected WT (WT inf) and infected IRF4-KO (IRF4-KO inf) mice were isolated. **a**, **b** Proportions of the CD19^+^ B cells in the liver, spleen and lung were evaluated by flow cytometry. Data are expressed as the mean ± SD of 3–6 mice. **c**-**d** Detection of CD69 expression in CD19^+^ B cells from the liver, spleen and lung by flow cytometry. Data are expressed as the mean ± SD of 3–6 mice. **e** Expression of IgG and IgM in the serum of infected mice was detected by ELISA. Asterisks indicate a statistically significant difference at *P < 0.05, compared with the corresponding control (unpaired *t*-test). ELISA, Enzyme-linked immunosorbent assay; FMO, fluorescence minus one; IgG/IgM, immunoglublin G/immunoglobulin M; IRF4, interferon regulatory factor 4; IRF4-KO, IRF4 knockout mice strain; IL, interleukin; SD, standard deviation; WT, wild-type

The investigation into the impact of IRF4 myeloid knockout on B cell activation and humoral immunity in mice infected with *S. japonicum* was carried out with a focus on the expression of CD69, a pivotal early marker of immune cell activation. Given its sensitivity and prompt upregulation upon antigenic stimulation or cytokine exposure, CD69 serves as a reliable indicator of B cell involvement in immune responses. To assess these dynamics, FCM was used to analyze the proportion of CD19^+^ B cells and assess the CD69 expression level of B cells in both WT and IRF4-KO mice infected with *S. japonicum*. In comparison to infected WT mice, no significant differences were observed in the proportion of CD19^+^ B cells in the liver, spleen and lung of infected IRF4-KO mice (Fig. 7a, b; *t*-test: WT inf versus IRF4-KO inf: Liver: *t*(6)=1.44, *P* = 0.1987; Spleen: *t*(8)=0.5417, *P* = 0.6028; Lung：*t*(6)=0.7927, *P* = 0.4581). Notably, however, the expression of CD69 in lung CD19^+^ B cells of infected IRF4-KO mice was significantly higher than that of in lung CD19^+^ B cells of infected WT mice (Fig. 7c, d; *t*-test: WT inf versus IRF4-KO inf: *t*(8)=2.551, *P* = 0.0341), while no significant difference was detected in the liver and spleen (Fig. 7c, d; *t*-test: WT inf versus IRF4-KO inf: Liver: *t*(7)=1.296, *P* = 0.2361; Spleen: *t*(8)=0.5243, *P* = 0.6143). To further investigate humoral immune responses, the levels of SEA-specific IgM and IgG in the serum of infected mice was measured by ELISA. The results indicated no significant difference in the IgG and IgM levels between infected WT and infected IRF4-KO mice (Fig. 7e; *t*-test: WT inf versus IRF4-KO inf: IgG: *t*(13)=1.215, *P* = 0.2459; IgM: *t*(13)=1.509, *P* = 0.1553), suggesting that IRF4 myeloid knockout does not significantly impact overall humoral immunity in mice infected with *S. japonicum*.

## Discussion

Schistosomiasis is an acute and chronic parasitic disease caused by *S. japonicum*, which seriously affects economic development and human health. MDSCs have been reported in autoimmunity, pregnancy, obesity and infectious diseases [37], but the effect of IRF4 on MDSCs in *S. japonicum* infection has been rarely reported. The results of our study provide evidence that myeloid knockout of IRF4 increases the immune response by reducing infection-induced MDSCs in *S. japonicum* infection, thereby alleviating the inhibitory effect of T cell proliferation and activation.

*Schistosoma japonicum* and *Schistosoma mansoni* can cause high-intensity infections (characterized by high worm burdens), resulting in a high incidence of disease [2, 38, 39]. SEA produced or shed by schistosome eggs can trigger an inflammatory immune response, including granulomas, which lead to diseases of the liver and spleen, as well as the intestinal and genitourinary tract. In the present study, the liver and spleen of *S. japonicum*-infected mice showed considerable enlargement, color deepening and weight gain (Fig. 1a). H&E staining revealed the presence of inflammatory cells infiltration in the liver and lung and a large number of granulomas in the liver (Fig. 1b). Granulomas are the most prominent pathological feature of schistosomiasis, and their number and area can reflect the degree of host infection [40, 41]. Our results showed that the area covered by granulomas in the liver was significantly reduced in IRF4-KO mice infected with *S. japonicum* compared to infected WT mice (Fig. 1c). Studies have reported that the levels of AST and ALT continue to increase in patients infected with *S. mansoni* [42]. Our results showed that the serum ALT level of IRF4-KO mice decreased significantly after infection with *S. japonicum* (Fig. 1d). All of these observations suggested that the lack of IRF4 during *S. japonicum* infection contributes to the mouse’s resistance to the parasite; however, the specific mechanism needs to be further explored. It is noteworthy that IRF4 has diverse functions in both T cells and myeloid cells. While prior studies have documented decreased T cell cytotoxicity and immunoglobulin production following IRF4 knockout in T cells [27], our investigation specifically focused on the consequences of IRF4 knockout in myeloid cells, particularly on MDSCs. The contrasting effects of IRF4 in the two MDSC cell types likely contribute to the intricate immune landscape observed during *S. japonicum* infection. By suppressing MDSCs, IRF4 knockout in myeloid cells may mitigate their immunosuppressive influence on T cells, subsequently facilitating increased T cell proliferation, activation and cytokine production. This, in effect, could compensate for the potential decline in T cell cytotoxicity and immunoglobulin production resulting from direct IRF4 knockout in T cells. Collectively, these findings underscore the intricate interplay between IRF4-mediated signaling across distinct immune cell subsets and their cumulative influence on the outcome of *S. japonicum* infection.

The term MDSCs was originally used to describe the heterogeneous immature myeloid cell population in the pathological environment [43], which has strong immunosuppressive activity and has been shown to be induced to expand and accumulate during helminth and protozoan parasite species infection [22]. In the present study, the percentage of MDSCs in the liver, spleen, lungs and BM of mice infected with *S. japonicum* significantly increased (Fig. 2a, b), which was consistent with the expansion of MDSCs in lymph nodes, lungs and BM of mice infected with *S. japonicum* [23]. In addition, the percentage of MDSCs in the liver, spleen, lungs and BM of *S. japonicum*-infected IRF4-KO mice was significantly lower than that the same organs of infected WT mice (Fig. 2a, b). This finding may imply that IRF4 plays an important role in regulating the migration of MDSCs from BM to other tissues. To further validate this possibility, we plan to trace the migration path of MDSCs by FCM in future studies and detect the expression of related chemokines and adhesion molecules.

Invasion of *S. japonicum* cercariae into the host skin leads to the activation of resident macrophages and DCs [37]. Classical DCs have been shown to be essential for inducing type 2 inflammation in the liver and spleen of mice infected with *S. japonicum* [44, 45]. Macrophages play a key role in granuloma formation and immune regulation, as well as a leading role in tissue repair during schistosomiasis [46, 47]. Our results showed that the percentage of DCs in the spleen and the percentage of macrophages in the liver, spleen, lungs and BM increased significantly after *S. japonicum* infection. After IRF4 myeloid knockout, the percentage of DCs in the liver of mice infected with *S. japonicum* decreased significantly, and the percentage of macrophages in BM decreased significantly (Fig. 2c-f). Some studies have shown that IRF4 plays a role in the development of monocytes, plasmacytoid DCs (pDCs) and classical DCs (cDCs) [48–50], contributing to the differentiation of pDCs [48]. However, our results show that IRF4 knockout cells had little effect on the percentage of DCs, possibly be due to the limited effect of knockout cells in mice. In *S. mansoni* infection, the consumption of CD11b^+^F4/80^+^ macrophages reduces the size of granulomas [47]. The authors of a previous study reported that the absence of IRF4 can reduce renal fibrosis after acute renal injury by reducing the recruitment and activation of macrophages [51]. Therefore, the deletion of IRF4 in *S. japonicum* infection may reduce the area of granuloma and reduce the damage of *S. japonicum* infection to mice by reducing the accumulation of macrophages.

There was no significant difference in the percentage of CD11b^+^Ly6G^+^ Ly6C^−/low^ PMN-MDSCs and CD11b^+^Ly6G^−^Ly6C^high^ M-MDSCs between infected WT mice and infected IRF4-KO mice in our study (Fig. 3a, b), indicating that myeloid-specific IRF4 knockout does not affect the distribution of MDSC subsets in *S. japonicum*-infected mice. As shown in Fig. 3c-e, the suppressive effect of MDSCs on T cell proliferation is reduced in infected IRF4 knockout mice, and this attenuation may be partially mediated by reduced expressions of *NOS2* and *p67*^*phox*^, but could also involve other unknown mechanisms.

In tumor and pathogen infection models, programmed death receptor ligand 1/2 (PD-L1/L2) can be expressed in myeloid cells, including MDSCs, and bind to PD-1 expressed on activated T cells to induce cytotoxic T cell (CTL) failure [52, 53]. As shown in Fig. 4a, b, we found that the deletion of IRF4 significantly reduced the percentage of PD-L2^+^ MDSCs in the liver of mice infected with *S. japonicum*, indicating that the deletion of IRF4 suppressed the immunosuppressive function of MDSCs induced by *S. japonicum* infection by downregulating the expression of PD-L2 in the liver of infected mice. In order to further explore the effect of IRF4 on inflammatory factors in MDSCs in mice infected with *S. japonicum*, we compared the percentage of IL-1α^+^, GM-CSF^+^, IL-6^+^ and IL-10^+^ cells in MDSCs of the liver, lung and spleen of infected mice. The results showed that IL-1α secreted by MDSCs in the liver, spleen and lung of IRF4-deficient mice was significantly lower than that of infected WT mice (Fig. 4c, d). qRT-PCR also showed that the expression of IL-1α in spleen MDSCs decreased significantly after myeloid loss of IRF4 (Fig. 4e, f). These results suggest that the deletion of IRF4 may weaken the inhibitory function of MDSCs in the liver, spleen and lungs of infected mice by reducing the secretion of IL-1α.

It has been reported that MDSC apoptosis can affect the accumulation of MDSCs [54]. Our results showed that compared with infected WT mice, the expression of annexin V in the spleen and BM of infected IRF4-KO mice decreased significantly (Fig. 5a, b), and that MDSCs in the liver, lung and BM of infected IRF4-KO mice also decreased significantly (Fig. 2a, b). This is contrary to the finding that knockdown of IRF4 in myeloma can induce apoptosis of myeloma cells, which may be caused by disease models and different cells [55]. The authors of other studies have reported that napabucasin, an inhibitor of STAT3, could eliminate the immunosuppressive ability of mouse MDSCs and human M-MDSCs, and improve the survival rate of mice carrying melanoma [56]. AKT1 kinase has been found to enhance the inhibitory function of MDSCs in cancer and inflammation [57]; in this investigation, p-STAT3 and p-AKT were activated. It has been discovered that the impact of IRF4 on the formation of MDSCs during *S. japonicum* infection is significantly influenced by the activation of STAT3 and AKT. These results help to clarify that the p-STAT3 and p-AKT pathways are involved in the amplification of MDSCs induced by *S. japonicum* infection.

Unlike other IRF family members, IRF4 is not induced by interferon, but is mainly regulated by TCR signal transduction, BCR, TLR and tumor necrosis factor receptor [27]. It has been reported that in chronic lymphocytic choriomeningitis virus infection, reducing the expression of IRF4 restores the function and metabolic characteristics of antigen-specific T cells and promotes the development of memory-like T cells [58]. In exploring the effect of myeloid cells on T cells after IRF4 myeloid knockout, we detected the percentage of T cells in liver, spleen and lungs of infected mice. Our results showed that the percentages of CD3^+^CD4^+^ T cells and CD3^+^CD8^+^ T cells in the liver, spleen and lungs of infected IRF4-KO mice were significantly higher than those in infected WT mice, but had no significant effect on T cell activation (Fig. 5a-d). The detection of cytokines showed that IL-4 and IFN-γ secreted by CD3^+^CD4^+^ T cells and CD3^+^CD8^+^ T cells in the lungs of infected IRF4-KO mice were significantly higher than those in infected WT mice (Fig. 5e-h). As shown in Fig. 2a, b, only the percentage of MDSCs in myeloid cells after IRF4-KO myeloid knockout in *S. japonicum* infection decreased significantly. We speculate that myeloid knockout in IRF4 will lead to a decrease in the proportion of MDSCs and a decrease in inhibitory function, which leads to the increase of T cells.

Antibody response plays an important role in *S. japonicum* infection, and B cells are usually thought to participate in the immune response by producing antibodies. Although B lymphocytes support the establishment of strong Th2 profiles associated with worm infection [59], they have recently been shown to play an active regulatory role in the process of schistosomiasis infection, mainly affecting T cell response [60]. At present, most of the experimental studies on B cells induced by *Schistosoma* are on *S. mansoni* [60, 61]. However, there are significant differences in immunopathogenesis and immunomodulation between *S. mansoni* and *S. japonicum* [41]. Our study showed that there was no significant difference in the percentage of B cells in the liver, spleen and lungs between infected WT mice and infected IRF4-KO mice, and that there was no significant difference in B cell activation and serum antibody expression between the liver and spleen (Fig. 7a-e). It is suggested that the decrease in the proportion and function of MDSCs caused by IRF4 myeloid gene knockout has no significant effect on the differentiation, activation and antibody production of B cells in the process of *S. japonicum* infection.

In summary, the results of our study demonstrate that IRF4 myeloid knockout can alleviate liver and lung damage in mice infected with *S. japonicum*. It also suppresses MDSCs by reducing PD-L2 expression and IL-1α secretion. Additionally, IRF4 myeloid-specific knockout promotes T cell activation and expansion but does not affect B cell aggregation and activation. These findings suggest that IRF4 regulates MDSCs and tissue damage during *S. japonicum* infection through the STAT3 and AKT signaling pathways, providing potential therapeutic targets for controlling this parasitic infection.

## Supplementary Information

Additional file 1: Figure S1. The proportion of liver, spleen, and lung weight in the total body weight.

## Data Availability

No datasets were generated or analyzed during the current study.
