# Supplementary material for: IRF4 regulates myeloid-derived suppressor cells expansion and function in Schistosoma japonicum-infected mice
Source: Parasit Vectors. 2024 Nov 28;17:492. doi: 10.1186/s13071-024-06543-8 (PMC11605884; doi:10.1186/s13071-024-06543-8)
Supplement: Supplementary file 1 — Additional file 1: Figure S1. The proportion of liver, spleen, and lung weight in the total body weight. [file 13071_2024_6543_MOESM1_ESM.docx]

**Supplementary Data**


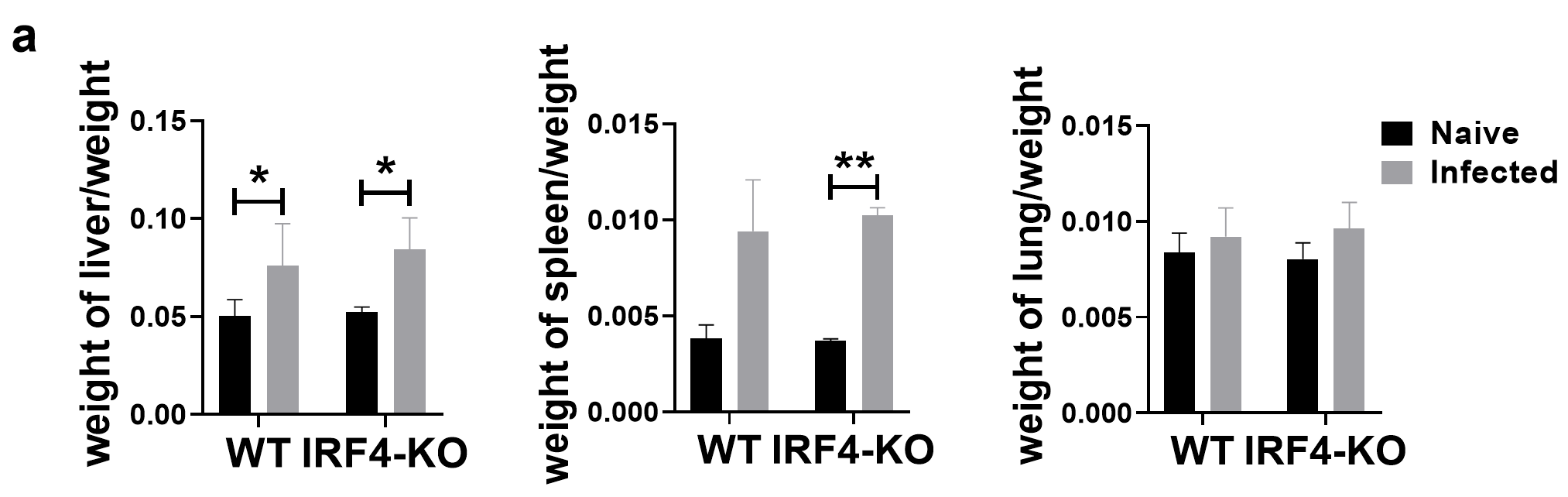


**Fig. S1** The proportion of liver, spleen, and lung weight in the total body weight. (a) WT mice and IRF4-KO mice were infected percutaneously with 40 ± 5 cercariae and sacrificed at 6-7 weeks after infection. The tissues of liver, spleen and lung were harvested. The weight of the liver, spleen, and lung in WT and IRF4-KO naive and infected mice was expressed as a percentage of the total body weight. Data are presented as mean ± SD of 3-12 mice. * *p*<0.05, ** *p*<0.01 compared with the corresponding control, unpaired *t*-test was used.
